# Knowledge-based best of breed approach for automated detection of clinical events based on German free text digital hospital discharge letters

**DOI:** 10.1371/journal.pone.0224916

**Published:** 2019-11-27

**Authors:** Maximilian König, André Sander, Ilja Demuth, Daniel Diekmann, Elisabeth Steinhagen-Thiessen

**Affiliations:** 1 Charité-Universitätsmedizin Berlin, Corporate Member of Freie Universität Berlin, Humboldt-Universität zu Berlin, and Berlin Institute of Health, Lipid Clinic at Interdisciplinary Metabolism Center, Berlin, Germany; 2 Charité-Universitätsmedizin Berlin, Corporate Member of Freie Universität Berlin, Humboldt-Universität zu Berlin, and Berlin Institute of Health, Department of Nephrology and Internal Intensive Care Medicine Berlin, Germany; 3 ID Information und Dokumentation im Gesundheitswesen GmbH, Berlin, Germany; 4 Charité - Universitätsmedizin Berlin, BCRT—Berlin Institute of Health Center for Regenerative Therapies, Berlin, Germany; Universitatsklinikum Jena, GERMANY

## Abstract

**Objectives:**

The secondary use of medical data contained in electronic medical records, such as hospital discharge letters, is a valuable resource for the improvement of clinical care (e.g. in terms of medication safety) or for research purposes. However, the automated processing and analysis of medical free text still poses a huge challenge to available natural language processing (NLP) systems. The aim of this study was to implement a knowledge-based best of breed approach, combining a terminology server with integrated ontology, a NLP pipeline and a rules engine.

**Methods:**

We tested the performance of this approach in a use case. The clinical event of interest was the particular drug-disease interaction “proton-pump inhibitor [PPI] use and osteoporosis”. Cases were to be identified based on free text digital discharge letters as source of information. Automated detection was validated against a gold standard.

**Results:**

Precision of recognition of osteoporosis was 94.19%, and recall was 97.45%. PPIs were detected with 100% precision and 97.97% recall. The F-score for the detection of the given drug-disease-interaction was 96,13%.

**Conclusion:**

We could show that our approach of combining a NLP pipeline, a terminology server, and a rules engine for the purpose of automated detection of clinical events such as drug-disease interactions from free text digital hospital discharge letters was effective. There is huge potential for the implementation in clinical and research contexts, as this approach enables analyses of very high numbers of medical free text documents within a short time period.

## Introduction

Increasing patient numbers and ever-shorter length of hospital stays, as well as growing multimorbidity and polypharmacy call for information technology solutions to achieve considerable improvements in the quality and efficiency of health care, especially with regard to the medication process. Indeed, the urgent need for automated tools that can improve health care processes, e.g. by providing real-time support in the medication process, is underlined by memoranda to this field.[1]

In the digital era, comprehensive medical information pertaining to a given patient are usually available in electronic medical records (EMR). These data, such as medical history, exam results, physician notes, and in particular hospital discharge letters, contain high-quality information, and therefore are a valuable resource which could be utilized to improve the quality of care (e.g. in terms of care quality assessment, disease surveillance, and adverse event detection), but also for research purposes.

However, medical data, and particularly discharge letters are usually unstructured and mostly written in free text. At present, patient records (electronic or paper-based) and discharge letters still have to be manually reviewed in order to retrieve the information of interest–particularly in view of large numbers of documents this is time-consuming, tedious, error-prone, or impossible at all. Therefore, what is missing is high-performing systems that can process, read and analyze medical free text documents in a highly automated manner.

Indeed, clinical narratives still present a huge challenge to available text analytics systems, most of which are based on natural language processing [NLP], since the medical terminology is extensive and very complex.[2, 3] With less complex sources, such as death certificates or billing information, such approaches have been successfully established. [4] [5] Also results of recent studies, which have dealt with more complex tasks, were promising. E.g. Iqbal et al. were successful in identifying antipsychotics and antidepressants-related adverse drug events (ADEs) from within the free text of psychiatric EMRs, albeit their approach was very specific to this particular study question [6–8] [9, 10].

In the last years, ontology-driven rule-based systems have shown very good results for information extraction tasks in various clinical domains.[11]

However, applications for non-English text, e.g. publications that have dealt with German-language applications are scarce, primarily due to restrictive data protection standards in Germany and Europe, impeding NLP research, as sharable, open-source language resources play a pivotal role for performance testing and classifier training. [12] Recently, e.g. Richter-Pechanski et al. showed the application of NLP on German texts with the goal of de-identification.[13] Another group of researchers from the University of Heidelberg used NLP technologies to extract diagnoses from German diagnostic reports[14].

The Medical Informatics Initiative by the German government has now led to the creation of a national reference corpus for German clinical documents be made accessible on an on-demand basis.[12] The same group of researchers also presented an approach of creating synthetic text corpora, which could overcome the limitation of availability[15]. In the introduction of their publication Lohr et al. presented a good overview on current German text corpora. Furthermore, they also recently presented an approach for de-identification, which might lead to more accessible data [16].

A very good overview on the current status of NLP on German texts is given in the review of Jungmann et al.[17] Especially temporal information extraction and the correct interpretation of intentionally vague indications of degrees of certainty pose largely unsolved challenges. [18]

Previous studies have mainly focused on the creation of algorithms to recognize events pertaining to one specific domain, which could be extracted successfully each with a specific approach (e.g. extrapyramidal side effects of drugs for mental illness).[10] In the majority of cases, these were NLP-based systems that mapped free text on a specific ontology, or systems, which detected input parameters by means of information extraction.[6,7,13] [10] While due to lack of appropriate resources, commonly to begin with refinement or de novo generation of a terminology have been necessary, we could recently demonstrate that analysis of free-text-containing medical records is possible with standard tools and terminologies. [19]

Despite innumerable possible applications (e.g. for quality assurance: sentinel systems that are able to perform medication safety checks using the information contained in free text in discharge letters) information extraction from clinical narratives has not been widely implemented into clinical routines.

Undoubtedly, there is a need for generic high-performing applications that are able to process large amounts of data within short periods of time [9, 20, 21]. We envision tools that are able to extract comprehensive sets of medical concepts from the given source (e.g. the electronic medical record) in order to perform complex quality or plausibility checks, such as matching drug prescriptions and diagnoses [22].

Thus, we aimed to develop a largely generic system, which can be easily adapted to deal with new text analytics tasks or types of clinical problems, without requiring substantial training or learning. We designed SemDrugS (Semantic Drug Surveillance) as a best-of-breed approach, using and combining established components, such as a natural language processing (NLP) pipeline, a rules engine and a terminology server.

We decided to use a rule-based approach over a model-based approach mainly because we aimed for a high sensitivity (recall). Some clinical events are very rare, which means there is almost no training data available. However, our system should be able to detect these rare cases. This also applies to rarely used active agents. Therefore, we conceived an approach, which can be implemented with little or even no training [19]

We aimed to test whether this knowledge-based approach is on a par with other state-of-the-art free text analysis tools, and assess its usefulness for clinical use. Therefore we conceived an exemplary use case. Using free text digital hospital discharge letters, cases with the specified drug-disease interaction (DDI) “proton-pump inhibitor use and osteoporosis”should be extracted. DDIs, which are adverse interactions between a drug and a disease or condition that a patient has, are common and they are difficult to track and detect without support by algorithms.

Use of proton pump inhibitors (PPI) is associated with reduction in bone mineral density[23]. Thus, PPIs should be avoided or used with caution (in terms of a relative contraindication)[24] in subjects with or at high risk of osteoporosis. This potential DDI is likely to be very common in elderly patients. In 2015, 13.4 million people in Germany were prescribed PPIs–i.e. about one in six inhabitants. Especially among older adults, the prevalence of use is very high.[25] Of note, the percentage of cases without adequate indication for the use of PPIs is large (>30%).[24, 26] PPIs are often prescribed for acute gastritis or during a hospital stay as stress ulcer prevention, but nevermore stopped, although the original indication has ceased to exist. In 2015 PPIs were added to the Beers’ list of potentially inappropriate drugs (PIM) for older adults.[27, 28] [23, 24, 29] It is important to note that osteoporosis and osteoporosis-related fractures are associated with high morbidity and mortality, as well as high health care costs.[19][20]

## Methods

### Data

We used discharge letters that were generated in the Berlin Aging Study II (BASE-II). In this epidemiological study, approximately 2,200 participants received an extensive baseline medical examination between 2009 and 2014.[30, 31] It was a special feature of this study that every participant got a discharge letter, summarizing the relevant medical findings that were made in the study. These letters were written in German language, in the style of a standard discharge letter from Charité-Universitätsmedizin Berlin, including a list of conditions and diagnoses, sections on diagnostic findings, laboratory values, medication, and a detailed discharge summary. All sections contained either unstructured free-text or syntactically semi-structured text. The overall structure (Table 1) of the documents varied considerably over the study period of five years. A brief analysis of the corpus is given in Table 2. An example of a discharge letter is provided in the supplement (S1 File).

**Table 1:** Basic structure of the discharge letters in the BASE-II study.

Age, Year of birth, Sex
New diagnoses
Previous diagnoses
Medication
Results of physical examination
Results of neurological examination
Blood pressure
Addiction: smoking, alcohol
Geriatric assessment
Adjuvants
Laboratory values
Electrocardiogram (ECG)
Pulse wave analysis
Dual Energy X-ray Absorptiometry (DXA)
Bioelectric impedance analysis (BIA)
Spirometry
Audiometry
Eye refraction test
Tonometry
Depression screening
Discharge summary

**Table 2:** Description of corpus.

Number of documents:	1,982
Total lines:	184,022
Average lines per document:	93
Total number of tokens:	2,001,114
Average tokens per document:	1,010
Number of unique tokens:	57,745
Average length of token:	11

### Preprocessing

The discharge letters were only in part available as digital text. For the rest (approximately n = 600), only a paper based version was available. Optical Character Recognition (OCR) was used (Adobe Acrobat Pro®) to transform the paperbased documents into digital text, retaining the original structure of the documents. Finally, all discharge letters were pseudonymised before further processing. Names, unique numbers and dates of birth were manually removed, in compliance with the HIPAA Safe Harbor method. [32] Only the study ID was kept to be able to assign participants to the gold standard.

After preprocessing, 1982 discharge letters were available for our analyses.

### The SemDrugS system

**Fig 1:**
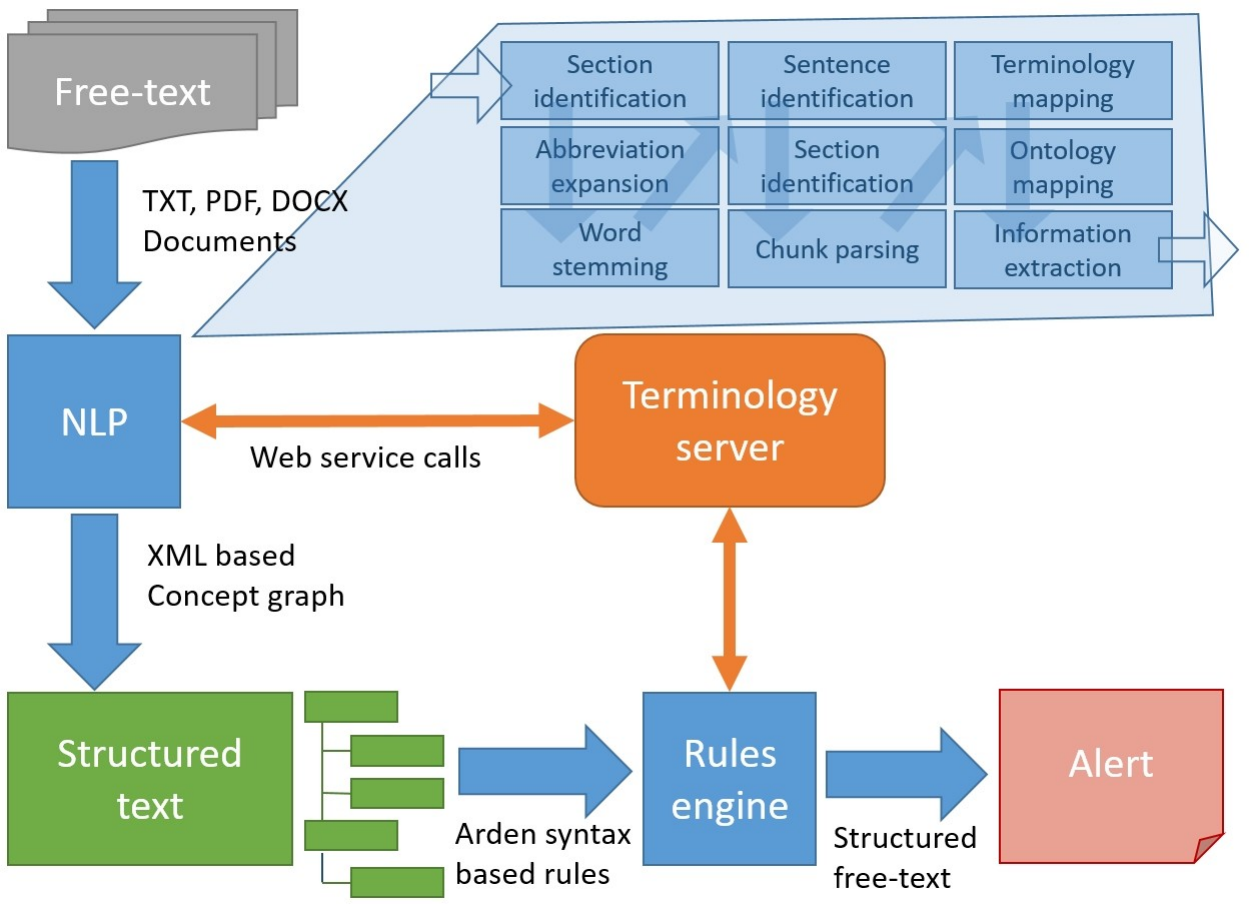
Architecture of the SemDrugS approach. All components used the terminology server and the included ontology in order to facilitate a semantic interpretation.

Fig 1 provides a schematic representation of the architecture of SemDrugS (Semantic Drug Surveillance). The approach, which is described in this paper, was composed of three main components:

- A terminology server with an integrated ontology provided access to the Wingert nomenclature (WNC) [27, 28], a comprehensive and precise terminology of the medical domain. Our architecture consumed the CTS2 Application Programming Interface (API). Alternatively any other compliant terminology server may be used [33].
- The NLP pipeline provided all components necessary for the processing of natural language. These include stemming (reducing words to their meaningful stem and splitting up compound nouns), parsing (breaking down a text into its component parts of language with an explanation of the form, function, and syntactic relationship of each part), expansion of abbreviations, disambiguation, and extensive spell-correction algorithms. The OCR processing added some errors with very typical patterns (e.g. “h” is recognized as “n” etc.) to the text. Recognizing such patterns is also one of the features of the NLP engine. The result of this multistep process is a machine-readable syntactic representation and interpretation of natural language. Our NLP engine used standard components based on GATE/JAPE as well as newly developed components, like a stemming algorithm, which is able to break up German compound nouns.[34] The concept identification was implemented within the NLP engine and contained concepts provided by the terminology server.
- The rules engine facilitated the definition and implementation of rule-based knowledge-modules. The definition was realized in Arden syntax [35], a computer-language, which is optimized for defining rules in the medical domain and enables phrasing, which is close to natural language. We used a commercial implementation provided by Medexter Healthcare [36].

### Implementation

We developed a software that applied the NLP engine to all discharge letters (one by one full-text), which were mapped onto the terminology, the Wingert nomenclature (WNC), during that process [37, 38].

The origin of the Wingert-Nomenclature (WNC) roots back to the mid 1980s when it started as a German translation of SNOMED 2. In the same way as SNOMED 2 evolved to SNOMED CT and became an ontology, the WNC terminology is now organized as an ontology and can be expressed in description logic (which was not used here, though). It is fully available (commercial license) in German (a required prerequisite for processing German free-text) and all relevant medical domains are covered, which include the following major categories: diagnoses, morphologies, treatments, procedures, agents, microbiology, function, materials. Especially the whole terminology from some major classifications like ICD-10, LOINC, ATC and the German Operations and Procedures Key (OPS) is contained in the Wingert Nomenclature, which is an important prerequisite to achieve uniform results for patients from diverse medical departments.

The WNC contains about 110.000 concepts with about 250.000 descriptions. Such descriptions are typically synonyms and related terms but also translations.

The relatively low number of descriptions per concept does not influence the clinical expressiveness because synonyms are also created virtually during the annotation process. This can be best explained by the following example: given a concept has two descriptions “heart” and “cardiac” (which are related but not synonymous terms), and given a second concept “heart infarction”. In this case the system recognizes the compound term “cardiac infarction” equal to “heart infarction”, because “heart” and “cardiac” are subsumed under the same concept, forming a “virtual synonym”. This approach is especially useful in languages using compound nouns, such as German and Dutch.

In the ontology all concepts are connected via taxonomic (“is a”), partonomic (“is part of”), and semantic relations like “is contraindication of”. E.g., a typical concept would be “M000562”, which contains 25 terms in seven languages: Inflammation, inflammatory process, inflammatory, inflammatory illness, etc.

The features of the NLP pipeline include fully-automated expansion of abbreviations (PPI and *proton-pump inhibitor* are detected identically), a disambiguation algorithm (which uses the ontology itself to resolve ambiguous terms, by exploring the context before and after the ambiguous term via semantic paths; if disambiguation is not possible, all possible interpretations are used in parallel), management of synonyms, as well as a spell-checker and a spelling-correction algorithm (e.g. „prtone-pmp inhbtor“). Moreover, the NLP engine is capable of detecting negations (e.g. “no signs of osteoporosis”). Furthermore, the NPL engine is able to access further databases, e.g. to resolve tradenames of drugs. Finally, a very helpful feature is the integration of a pattern matching system, providing the basis for regular expression detection and named entity recognition (NER). This feature is important to recognize and extract laboratory values and results of clinical measurements, e.g. blood pressure measures. Also, some discharge letters contained ICD-10 codes, which were extracted by means of regular expressions. Following their extraction, the textual label of each code is looked up in the terminology and fed back into the NLP engine to be mapped onto the Wingert nomenclature. For example, the ICD-10 code “M82.02” is resolved to the label “Osteoporosis in multiple myelomatosis: Upper arm”, which is then mapped to “M0006E0 Osteoporosis T000439 upper arm GA00026 in M000E3C multiple myelomatosis”. The use of ICD-10 codes when given in the document is to be favored over free-text since a unique label is assigned to every ICD-10 code.

Bone densitometry results were identified by means of NER, using the keyword “T-Score”(“name”). The corresponding concept id in the terminology is “W000F9D”. The corresponding measured value was retrieved and processed via the integrated rules-engine. The respective rule was implemented to identify both osteoporosis as well as osteopenia, according to the WHO definition (S1 Table). The outcome of the rule, i.e. the interpretation of the bone density measurement, was added as a medical concept to the results of the NLP pipeline. For example the expression “T-Score: -2.7” led to the concept “M0006E0 Osteoporosis”.

With regard to medications, active agents, as well as trade names were detected and processed. Notably, the NLP pipeline identified drug trade names already on the level of the syntactic analysis. Stemming was applied to every single word, the result being pure word stems without suffixes. Subsequently, all stems were labeled with specific linguistic information, e.g. “trade name”. The conjunction of the trade names and active agents was then achieved by browsing a database of all drugs, that are available in Germany[39], looking up any stem that was labeled as “trade name”. Once the active agent had been identified, the corresponding concept in the terminology was retrieved. Notably, common trade names (e.g. “Aspirin”) were already included in the terminology.

**Fig 2:**
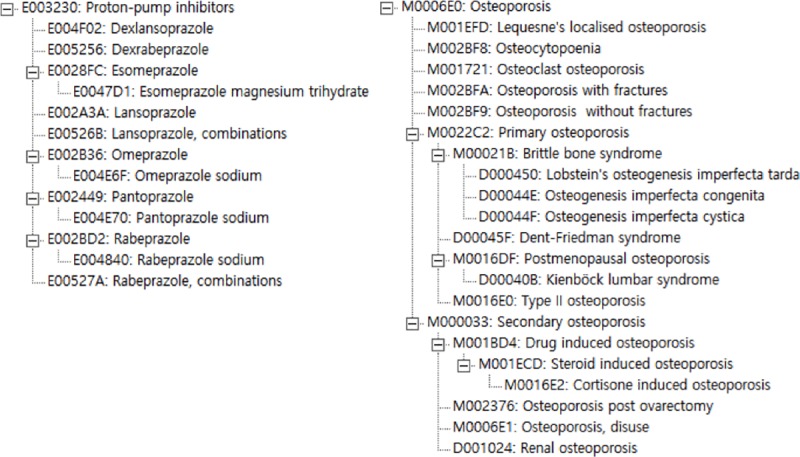
Hierarchical representation of osteoporosis and PPI in the ontology.

The ontology contained taxonomic relations. This facilitated the use of parent concepts instead of enumerations. To illustrate this, in Fig 2 the parent concepts “osteoporosis” and “PPI” and their descendants (subclasses) are represented.

The use of parent concepts allowed for a very compact formulation of rules, only referring to such concepts instead of using regular expressions or enumerations of string literals. In the present use case, we explicitly included only two concepts, the parent concepts “osteoporosis” and “PPI”, while there were 21 osteoporosis and 13 PPI subclasses (i.e. 273 possible combinations).

The output of NLP pipeline and terminology mapping was a xml based structure–a so-called “concept graph”–representing the underlying (linguistic) syntax and semantics. Finally, a rule was formulated in Arden syntax, linking the semantic concept “PPI”with the concepts “osteoporosis” and”osteopenia“, and defining this combination as a relative contraindication.

The data slot of the rule reads as follows:

trigger: = event {E003230}; /* PPI */

has_Osteoporosis: = call hasIndex with "M0006E0";

has_Osteopenia: = call hasIndex with "M002BF8";

The trigger event points to the parent concept “PPI” and thus all concepts semantically subsumed under “PPI” trigger the execution of that rule. Furthermore, the curly-braces-expression “hasIndex” also uses the taxonomy of the WNC to decide if one of the concepts found in the discharge letter is subsumed under either a “osteoporosis” or a “osteopenia” concept.

### Statistics

The results of the automatic DDI detection were compared to the preexisting study database of the BASE-II study. Since the generation of discharge letters was based on the same data, that had also been entered in the database, we could use this database as our gold standard.

After a manual review of all false positives and false negatives the precision, recall and F-score were calculated.

### Ethical considerations

The Berlin Aging Study II was performed in compliance with the World Medical Association Declaration of Helsinki on Ethical Principles for Medical Research Involving Human Subjects. The BASE-II study was approved by the Charité-Universitaetsmedizin Berlin ethics committee (approval number EA2/029/09). In addition to this no further ethics review was required for this particular analysis.

## Results

### Prevalence of osteoporosis and PPI use in the BASE-II study

According to our gold standard overall 1332 of the 1982 participants (67.2%) had evidence of osteoporosis or osteopenia, and 148 participants used a PPI (7.5%).

### Results of the automated detection and consistency with the gold standard

In 1298 out of 1982 participants (65%) SemDrugS found evidence of osteoporosis or osteopenia, and in 145 participants (7.3%) SemDrugS detected a PPI medication. Ninety-one participants were found to have the clinical event of interest, PPI use and osteoporosis/osteopenia (4.6%). Precision, recall, and F-score are provided in Table 3.

**Table 3:** Evaluation of the automated extraction versus gold standard.

	Osteoporosis/osteopenia	PPI	Osteoporosis/osteopenia and PPI
**True positives**	1298	145	87
**False positives**	80	0	4
**False negatives**	34	3	3
**True negatives**	570	1834	1888
**Recall (%)**	97.45 (96.45–98.23)	97.97 (94.19–99.58)	96.67 (90.57–99.31)
**Precision (%)**	94.19 (CI 92.42–95.58)	100.00 (99.50–100.00)	95.60 (93.92–96.84)
**F-Score (%)**	95.79	98.98	96.13

Notes: Data are given as N, or proportion and 95% confidence interval; N = 1982

## Discussion

The presented approach of combining a terminology server, a NLP pipeline, and a rules engine proved to be very effective. The clinical event examined was extracted with excellent precision and recall in a large set of free text discharge letters.

In order to extract the given DDI it was necessary that the two concepts “osteoporosis” and “PPI” were reliably identified. Particularly osteoporosis and osteopenia were only inconsistently listed in the diagnosis section, thus the necessary information had to be retrieved from measured values provided or by detection of a range of synonymous expressions, like “low bone density” in the narrative summary.

The number of participants that were recognised as osteoporosis-positive was higher than the true cases of osteoporosis as defined by the gold standard. Indeed in many discharge letters there were general recommendations for prevention of osteoporosis, with the term osteoporosis being mentioned when the doctor assumed an increased risk of osteoporosis, e.g. because of a borderline-result of the DXA, low vitamin D levels in the blood or other markers of bone breakdown (e.g. increased levels of deoxypyridinoline in the urine), but without an actual diagnosis of osteoporosis or osteopenia. Consequently, there were a considerable number of false positives. This is reflected by the suboptimal precision of 94.19% for the detection of osteoporosis.

Thus, although the system is already capable of detecting negations and a range of modalities, like speculations/conjecture, it proved to be problematic that the system is not yet capable of distinguishing between random general recommendations made, e.g. for a healthy diet for prevention of osteoporosis in cases with neither osteoporosis nor osteopenia, and true cases, where such a recommendation was justified by a given diagnosis or an increased risk of osteoporosis.

In contrast, both precision and recall were very high for the detection of PPI medication. There were no false positives and in only three cases, subjects with PPI were misclassified as not having a PPI (false negatives). Manual review showed that in two of these cases OCR quality was especially bad and in one case the prescription read “gastric acid protection”, which could not be interpreted as “PPI”.

Overall, with both precision and recall higher than 95%, the approach, which has been used in the present study, proved feasible and effective. It even outperforms current machine learning approaches, including implementations using modern neural network architectures, like Recurrent Neural Networks (RNN) und convolutional neural network (CNN). [40, 41]

Since the output of NLP pipeline, the concept graph, is a generic structure representing the syntax and the underlying semantics, only the rules have to be reformulated to adapt our approach to any use case. Such rules can combine any number of conditions and medications, as well as other entities like laboratory values, in an arbitrary boolean expression.

Notably, in contrast to other similar approaches [6,7], no curation of the terminology was required, since all concepts were already included.

As mentioned above, the use of terminology-based rules contributed to the excellent results, since all concepts were recognized equally and independent of their frequency. This is important to note, when comparing our approach to artificial intelligence-based algorithms, that always need a sufficient number of training data. In the presented approach training data is only needed for the annotation pipeline of the terminology server, which however can be regarded as a separate task. The terminology server that we used already had a very high annotation quality. [13, 19]

In contrast to previous studies, which have mapped free-text on a classification, [42, 43] e.g. the international classification of diseases (ICD-10), we instead used a comprehensive terminology and ontology. Any classifications have limitations when used for other than its intended purposes. E.g. ICD-10, the most widely used classification of diagnoses, is used worldwide mainly for morbidity and mortality statistics, and reimbursement systems, but only the upcoming ICD-11 revision will have a novel architecture that will allow a wider use. Drugs are not yet included in the ICD-10, but are most commonly classified according to the Anatomical Therapeutic Chemical Classification System (ATC) system. Thus for the given task several classifications would have been needed. Instead of using multiple classifications, we used the WNC terminology, which covered all relevant concepts contained in the discharge letters.

To the best of our knowledge, there is no directly comparable study. There are similar studies with English clinical text as mentioned in the above related work section [6–8] [9, 10]. E.g. the most readily comparable study by Iqbal et al. showed F-Scores of > 0.85 [6]. Admittedly, results based on different languages are difficult to compare, since every language is different, e.g. semantic concepts are different, and the different underlying structural rules (syntax, grammar, etc.) require different approaches for every language. Therefore NLP frameworks available for English language cannot readily be adapted to deal with German text.

In the foreseeable future, with the availability of sharable German corpora, also there will more and better possibilities of comparison. [12, 15]

This study demonstrates that the approach presented here is clearly feasible and can be of great value in medical practice and in research as it facilitates the processing of medical and pharmaceutical questions in a very efficient manner. Implementation into the hospital information system, e.g. via an alert function, could assist the user by means of a warning message in dedicated individual cases, while the background analysis of medical documentation does not add any additional burden to the healthcare professional.

Likewise, epidemiological studies could profit from the technology, which may ease, e.g. the identification of suitable patients with the exposure or outcome of interest to be included in their samples. In a similar vein, Cui et al. have already developed a query interface to be used in their research project for patient cohort identification [10].

We believe that combining established components in terms of a best of breed approach increases the robustness of semantic recognition, which is of crucial advantage, particularly when OCR quality is bad.

The very low number of “hits”in this use-case further illustrates the value of such technology, which is able to extract rare cases very efficiently (and with an precision higher than 95%), compared to the otherwise necessary manual review of thousands of documents, which is a time-consuming, tedious, and error-prone process.

### Strengths and limitations

A major advantage of this study was that the BASE-II study database provided a reliable gold standard for the validation of the results of the automated detection.

The discharge letters, we have used in this analyses were generated in an epidemiological study. Discharge letters from clinical practice may be more heterogeneous and thus our approach still has to prove its worth in clinical practice.

## Conclusion

We could show that our approach of combining NLP, a terminology server and a rules engine for the purpose of automated detection of a specified clinical event (e.g. drug-disease interaction) based on digital hospital discharge letters was effective. The good performance can be attributed to a comprehensive terminology, a well-structured ontology and a good annotation algorithm mapping the free-text onto the terminology.

Certainly, recent innovations and future developments in text mining technologies, particularly implementation of modern embedding techniques could help to further enhance the performance of the presented approach and similar approaches. [44, 45]

Knowledge-based systems show great promise for both clinical and research applications, as they facilitate effective analyses of very high numbers of medical text documents within a short time.

## Supporting information

S1 File Example of BASE-II discharge letter. (PDF)

S1 Table World Health Organization definition of osteoporosis based on bone density levels, WHO working group 1994. (PDF)
