# Supplementary material for: Knowledge-based best of breed approach for automated detection of clinical events based on German free text digital hospital discharge letters
Source: PLoS One. 2019 Nov 27;14(11):e0224916. doi: 10.1371/journal.pone.0224916 (PMC6881027; doi:10.1371/journal.pone.0224916)
Supplement: S1 File — (PDF) [file pone.0224916.s001.pdf]

CHARITÉ

A+P

ChariteCentrum für Innere Medizin mit Kardiologie, Gastroenterologie, Nephrologie

Charitß I Campus Virchow-Klln!kum 1 13344 Berlin

Forschungsgruppe Geriatrie
Lelierin: Prol. Dr. med. Mustermann

Berlln&r Allersstudie II
Projekt!eltung:
Prof. Dr. med. Mustermann

Unser Zeichen:
Tel. 030 450-553717
Fax 030 450-553947
base@egzb.de

Herrn
Dr. med. Mustermann
Facharzt für Allgemeinmedizin
Berlinstr. 1
12345 Berlin

Berlin, den 20.05.2012

Sehr geehrter Herr Dr. Mustermann,

wir berichten über Ihren Patienten Herrn xxxx , geb. 1938, der sich im Rahmen der Berliner Altersstudie Teil 2 (BASE W) bei uns am 15.05.2012 und 20.05.2012 vorstellte.

Vorliegende Diagnosen:	ICD 10
Vorhofflimmern	I48.19
Hypertonie	I10.90
BPH	N40
Arthrose	M19.09
Z.n. Synkope (2004)	R55
Z. n. Leistenbruch-OP (2001)	K40.3

Zusätzliche Diagnosen:

Hypercholesterinämie
Anämie
Osteopenie
Abnorme Befunde der Urin
Abnorme Befunde der Blutchemie

Aktuelle Medikation:

Acetylsalicylsäure	100 mg	1-0-0-0
Hydrochlorthiazid	25 mg	1-0-0-0
Metoprolol	50 mg	1-0-0
Tamulosin	0,4 mg	1-0-0-0
Ramipril	10 mg	1-0-0-0
Lercanidipin	5 mg	1-0-0-0

Körperlicher Status vom 15.05.2012:

71-Jähriger Studienteilnehmer in gutem Allgemeinzustand und gutem Ernährungszustand (Körpergröße 175 cm, Körpergewicht 87,0 kg, BMI 28,4 kg/m^2).
Haut: unauffällig, feuchte Schleimhäute, keine Ödeme, aktuell keine Wunden. Hals, Schilddrüse: keine obere Einflussstauung, keine Struma tastbar, gut schluckverschieblich. Cor: Herztöne rein, pulssynchron, keine Geräusche. Pulmo: sonor. vesikuläres Atemgeräusch ubiquitär. Abdomen: weiche Bauchdecken, keine Abwehrspannung, keine Resistenz palpabel, Peristaltik über allen Quadranten regelrecht, Leber unter Rippenbogen tastbar, Milz nicht tastbar. Nierenloge: bds. nicht klopfdolent. Lymphknoten: unauffällig. Bewegungsapparat: unauffällige WS, WS nicht klopfdolent, sämtliche Gelenke aktiv und passiv gut beweglich. Gefäßstatus: alle Arterienpulse bds. gut palpabel. Neurologischer Status: groborientierend unauffälliger Himnervenstatus, Pupillen isocor und lichtreagibel, Muskeltonus unauffällig, BSR, BRR, PSR und ASR jeweils symmetrisch, mittellebhaft. FNV metrisch, Babinski negativ, Trömmer-Reflex fehlt, Romberg-Versuch/Unterberger-Tretversuch: unauffällig.

RR im Stehen: 125/96 mmHg (rechts), 128/97 mmHg (links); HF 78 / min irrhythmisch,
RR im Sitzen:115/99 mmHg (rechts), 120/86 mmHg (links); HF 69 / min irrhythmisch,
RR im liegen: 120/83 mmHg (rechts), 117/89 mmHg (links); HF 61 / min irrhythmisch.

Geriatrisches Assessment:

Barthel-Index:	100 / 100 Punkte
IADL, Lawton/Brody:	8 / 8 Punkte
Tinetti-Test:	28 / 28 Punkte
Timed "Up-and-Go":	
MNA:	
GDS:	3 Punkte
ClockCompletionTest:	3 Punkte
DemTect:	18 / 18 Punkte
MMSE:	21 /30 Punkte

Befunde:
Die ausführlichen Befunde liegen in Kopie bei.

Labor vom 17.05.2010:
Auffällig waren:

Apoprotein A1 203 mg/dl, Zink 11,56 µmol/l, Gamma-GT 74,0 U/l, Bilirubin, ges. 104 mg/dl, (Bilirubin direkt und indirekt fehlten), Cholesterin ges. 253 mg/dl, LDL-Cholesterin 157 mg/dl, Magnesium 0,73 mmol/l, DHEA-Sulfat 5,2 nmol/l, Beta-Globulin (SPSP) 14 %, Erythrozyten 4,27 T/l, Hämoglobin 13,6 g/dl, Urin Mikroalbumin 78 mg/l

Oraler Glukosetoleranztest vom 17.05.2010: Keine gestörte Glukosetoleranz

Ruhe – EKG vom 17.05.2010: Vorhofflimmern, Hf 73/min, ansonsten unauffälliges EKG

Knochendichtemessung (DXA) vom 17.05.2010:
Normale Mineralisation im Bereich der LWS (T-Score 0,0; Z-Score 1,2)
Osteopenie im Bereich der linken Hüfte (T-Score -1,1; Z-Score -0,2)

Spirometrie: unauffällig.

Audiometrie: Hochton Hörverlust, bds.

Fernvisus:	rechts 0,5	links 0,25	(korrigiert)	rechts 0,5	links 1,0
Nahvisus:	rechts 0,2	links 0,1	(korrigiert)	rechts 0,6	links 0,7

Epikrise:

Im Rahmen der medizinischen Untersuchungen sahen wir einen Studienteilnehmer in gutem Allgemeinzustand. Von der Studie fielen die folgenden Befunde auf:

1. Die Kreislaufparameter waren im gesamten Verlauf stabil. Der bestehende Bluthochdruck ist medikamentös gut eingestellt aber laborchemisch zeigten sich erhöhte Gesamt· Cholesterin-und LDL-Werte als zusätzliche Risikofaktoren zur arteriellen Hypertonie für kardiovaskuläre Folgeerkrankungen. Aus diesem Grunde empfehlen wir die Einleitung einer medikamentösen Therapie mit LDL-Zielwert unter 100 mg-dl sowie die weitere regelmäßige Laborkontrolle der Lipidparameter.

2. Laborchemisch zeigten sich auffällige Hämoglobin und Bilirubin. Leider fehlten andere relevante Laborwerte. Möglichweise könnte die Anämie mit seiner Acetylsalicylsäure-Behandlung in Beziehung stehen. aber bei dieser Analyse gibt es zu wenige Informationen um diese Diagnose festzustellen. Wir empfehlen eine weitere Kontrolle dieser Parameter.

3. Bei der Knochendichtemessung konnte eine Osteopenie im Bereich der LWS festgestellt werden. Leider fehlte der Vitamin D-Wert. Zur Stärkung des Bewegensapparates sowie zur Osteoporose-prophylaxe wird eine calciumreiche Ernährung geraten.

4. Laborchemisch fiel ebenfalls ein Magnesium-Mangel auf. Eine orale Substitution von Magnesium ist wahrscheinlich empfehlenswert.

5. In der Audiometrie konnte eine Hochtonhörverminderung beidseitig nachgewiesen werden. Eine Wiederholung der Audiometrie sollte in ca. 1 bis 2 Jahren erfolgen.

6. Laborchemisch fielen Apoprotein A1, Zink, Gamma-GT; DHEA- Sulfat 5;2 nmol/l; Beta Globulin und Urin Mikroalbumin auf. Die hier auffällig gemessenen Laborwerte bitten wir erneut zu kontrollieren.

Die ausführlichen Befunde senden wir Ihnen in Kopie bei. Bei Rückfragen stehen wir Ihnen gerne zur Verfügung. Sollte es für Sie von Nutzen sein, können wir die folgende medizinische Versorgung anbieten:

Sprechstunde für Altersmedizin im Interdisziplinären Stoffwechselzentrum (Charite, Virchow-Klinikum, Augustenburger Platz 1, 13353 Berlin, Tel. 450-553 169, *stoffwechselcentrum@charite.de)*

LIpidambulanz des Interdiziplinären Stoffwechselszentrum (Charite, Virchow-Klinikum. Augustenburger Platz 1, 13353 Berlin, Tel. 450-553 169, *lipidambulanz@charite.de)*

Wir danken Ihnen für Ihre freundliche Mitarbeit und verbleiben mit kollegialen Grüßen.

Leitung der Berl' er Altersstudie II — Projektarzt

Anlagen:
Befund der DEXA-Messung
Befund der BIA-Messung
Spirometriebefund
Tonaudiogramm
Laborbefunde

• BASE-II ist ein gemeinsames Projekt der Forschungsgruppe Geriatrie der Chante am Evangelischen Gertatriezentrum, dem Max-Planck-Institut für Bildungsforschung, dem Max-Planck-Institut für molekulare Genetik und dem Sozio-Ökonomischen Panel (SOEP). In dieser von der Max-Planck-Gesellschaft und dem BMBF geförderten multidisziplinären Studie werden 2200 Probanden aus Berlin in zwei Gruppen (20-30 sowie 60-70 Jahre) untersucht. Mit besonderem Augenmerk auf die ältere Gruppe widmet sich BASE-II der Erfassung, dem Vergleich sowie dem Follow-up von medizinischen, kognitiven, neuropsychologischen, sozialen und ökonomischen Aspekten in den verschiedenen

Altersgruppen.
