# Supplementary material for: Knowledge-based best of breed approach for automated detection of clinical events based on German free text digital hospital discharge letters
Source: PLoS One. 2019 Nov 27;14(11):e0224916. doi: 10.1371/journal.pone.0224916 (PMC6881027; doi:10.1371/journal.pone.0224916)
Supplement: S1 Table — (PDF) [file pone.0224916.s002.pdf]

S1 Table. World Health Organization Definition of Osteoporosis Based on Bone Density Levels, WHO Working Group 1994. [39]

Level	Definition
**Normal**	Bone density is within 1 SD (+1 or −1) of the young adult mean.
**Low bone mass/Osteopenia**	Bone density is between 1 and 2.5 SD below the young adult mean (−1 to −2.5 SD).
**Osteoporosis**	Bone density is 2.5 SD or more below the young adult mean (−2.5 SD or lower).
**Severe (established) osteoporosis**	Bone density is more than 2.5 SD below the young adult mean, and there have been one or more osteoporotic fractures.

Notes: SD, standard deviation
